# Emergence and evolution of the renin–angiotensin–aldosterone system

**DOI:** 10.1007/s00109-012-0894-z

**Published:** 2012-04-14

**Authors:** David Fournier, Friedrich C. Luft, Michael Bader, Detlev Ganten, Miguel A. Andrade-Navarro

**Affiliations:** 1 Max-Delbrück Center for Molecular Medicine Berlin-Buch, Robert-Rössle-Str. 10, 13125 Berlin, Germany; 2 Charité–Universitätsmedizin, Berlin, Germany; 3 Experimental and Clinical Research Center, Berlin, Germany

**Keywords:** Evolution, Evolutionary medicine, Renin, Angiotensin, Aldosterone, Volume regulation, Salt, Hypertension, Cardiovascular diseases, RAAS

## Abstract

The renin–angiotensin–aldosterone system (RAAS) is not the sole, but perhaps the most important volume regulator in vertebrates. To gain insights into the function and evolution of its components, we conducted a phylogenetic analysis of its main related genes. We found that important parts of the system began to appear with primitive chordates and tunicates and that all major components were present at the divergence of bony fish, with the exception of the Mas receptor. The Mas receptor first appears after the bony-fish/tetrapod divergence. This phase of evolutionary innovation happened about 400 million years ago. We found solid evidence that angiotensinogen made its appearance in cartilage fish. The presence of several RAAS genes in organisms that lack all the components shows that these genes have had other ancestral functions outside of their current role. Our analysis underscores the utility of sequence comparisons in the study of evolution. Such analyses may provide new hypotheses as to how and why in today's population an increased activity of the RAAS frequently leads to faulty salt and volume regulation, hypertension, and cardiovascular diseases, opening up new and clinically important research areas for evolutionary medicine.

**Electronic supplementary material:**

The online version of this article (doi:10.1007/s00109-012-0894-z) contains supplementary material, which is available to authorized users.

## Introduction

Multicellular organisms regulate their internal environment by either modifying the extracellular volume or the solute concentration (osmolality). In mammals, the kidney is the final common pathway for both of these functions. Low- and high-pressure baroreceptors in various circulatory beds, as well as ion channels and sensors in the distal tubule, infer the body's volume. Ion channels in the hypothalamus sense the sodium concentration and extracellular fluid osmolality. Glomerular filtration rate, physical forces along the nephron, the sympathetic nervous system, and the renin–angiotensin–aldosterone system (RAAS) are the volume effectors. Osmolality effectors include thirst stimulation, release of the anti-diuretic hormone vasopressin, and the placement of aquaporin water channels in the renal collecting duct so that concentrated urine can be made. This study focuses on volume regulation. The RAAS is the principal volume-regulatory effector in mammals. It is a major regulator of blood pressure within the human body. Therefore, the RAAS has important implications for study of hypertension and other cardiovascular diseases [1].

**Fig. 1:**
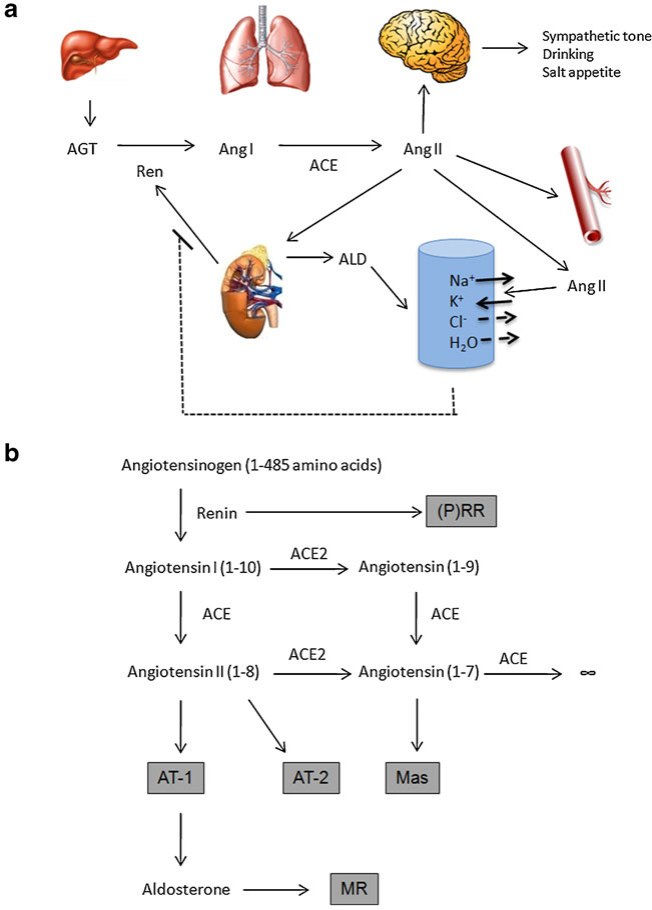
Scheme of the components of the hormonal RAAS. **a** Liver-produced angiotensin (AGT) is cleaved by renin from the kidney to the decapeptide angiotensin I (Ang I), which in turn is converted to Ang II (largely in the lung). The effector Ang II directs the adrenal gland to release aldosterone (ALD), which directs the brain to increase sympathetic tone, drinking, and salt appetite and also increases vasomotor tone. ALD, sympathetic tone, and Ang II act independently to affect NaCl reabsorption in the kidney. A reverse feedback mechanism exists. **b** The components involve a series of proteins (substrates, enzymes, and products) that can be defined by genomic study and followed across evolution. *(P)RR* prorenin receptor, *ACE* and *ACE2* angiotensin converting enzymes 1 and 2, *AT*_*1*_ and *AT*_*2*_ angiotensin receptors 1 and 2, *Mas* Mas receptor, *MR* mineralocorticoid receptor. In addition to this hormonal RAAS, the system also acts locally at the tissue level, e.g., brain, adrenal gland, and heart

The key effector precursor molecule of the RAAS is angiotensinogen, a protein produced principally in liver (Fig. 1a). Angiotensinogen is cleaved to a 10 amino-acid peptide, angiotensin (Ang I), by a unique aspartyl protease called renin, which is produced by the juxtaglomerular apparatus (JGA) in the kidney [2, 3]. The angiotensin-converting enzyme (ACE) in turn cleaves Ang I to a smaller, highly active 8 amino-acid peptide, angiotensin II (Ang II). ACE is a matrix metalloproteinase that is particularly highly concentrated on pulmonary endothelial cells, despite the fact that numerous functions of the enzyme in other bodily tissues besides the vasculature have been uncovered by elegant studies using genetically modified mice [4]. Moreover, other enzymes exhibit ACE-like activity, like the chymase, a protein mainly found in mast cells, which mostly displays such an activity in the heart [5]. Ang II acts on the adrenal cortex to release aldosterone (ALD). ALD acts in the kidney, primarily on collecting duct cells to effect reabsorption of sodium (and chloride). Ang II also has its own independent sodium reabsorptive effects in the kidney, acts in brain to stimulate thirst and salt appetite, as well as to increase sympathetic tone, and acts directly on the vessel wall (primarily arterioles) to affect vasoconstriction and to increase blood pressure [6]. Renin is the rate-limiting step in Ang II production. Renin release is stimulated by baroreflex mechanisms in the JGA, by beta-adrenergic sympathetic innervation of these cells, and by solute delivery, notably chloride content in the tubular fluid at the macula densa segment of the distal tubule. A negative feedback loop inhibits renin release that includes Ang II levels, inactivated baroreflex sensors, and sympathetic inhibition.

The RAAS involves a series of proteins working in a network (Fig. 1b). Renin, and its precursor molecule prorenin, can occupy a recently cloned receptor called the prorenin receptor (P)RR that binds both renin and prorenin [7]. The (P)RR can signal via MAP-kinase pathways and serves to activate prorenin. Its more global function is currently being investigated; however, the (P)RR has not been found to be implicated in volume regulation. Ang I is inert, while Ang II signals through two receptors: the AT_1_ and the AT_2_ receptors that have different functions, AT_1_ displaying two different isoforms (AT_1A_ and AT_1B_), which have been characterized in mice [8]. Ang I and Ang II can be cleaved to further products melding into a septapeptide called Ang (1-7). The recently discovered angiotensin-converting enzyme-2 (ACE2) is responsible for this cleavage activity. Ang (1-7) can signal through a unique receptor encoded by the *Mas* protooncogene, the Mas receptor. The steroid hormone ALD, stimulated by Ang II, signals through the mineralocorticoid receptor (MR).

In very general terms, the salt reabsorptive and vasoconstrictor mechanisms are stimulated by Ang II and are mediated by the AT_1_ receptor. The AT_2_ receptor has more ameliorative and modulating effects. The primary salt reabsorptive mechanisms are stimulated by ALD via the MR. Ang (1-7) appears to have actions that are generally opposite to those of Ang II so that its actions may also be ameliorative. The complex actions of the RAAS on its target organs and cellular messenger systems have been reviewed [6, 9].

## Evolution of the renin–angiotensin system

The evolution of the RAAS can be used to gain insights about its molecules and components, for which some questions remain open: Do we know all components of the system? Are they all similarly important? When did this system emerge and why? Is the system conserved in diverse animal species? Answering these questions is relevant to complete our understanding of how the system functions in humans, how it is involved in cardiovascular diseases, and why pharmacological interference of the RAAS is effective in their treatment [9, 10].

Today, genomic sequence data can be used to study RAAS evolution; however, the RAAS has been studied with comparative physiological techniques that also provided evolutionary insights. Homer Smith, who dominated renal physiology in the first half of the twentieth century, was extremely interested in comparative physiology and renal evolution [11]. He described the ureatelic phenotype of the lungfish, which can survive in mud-caked enclaves for over a decade without water. His activities culminated in a seminal book, *From Fish To Philosopher*, published in 1953 [12]. In a prescient fashion that would have pleased Claude Bernard, Smith describes the evolution of the *milieu intérieur* regulation in vertebrates. Smith followed renal evolution with model organisms from the present and what he could discern from fossil records. His notion was that organisms evolved in “relatively” brackish water with electrolyte contents similar to the extracellular environment of today. Evolution offered challenges, such as return to the sea and movement to fresh (electrolyte poor) water, to land, and even to the air. Organisms were faced with the problem of maintaining their internal environment and also excreting the end products of protein metabolism. Smith was interested in the different solutions that species have evolved for volume regulation and nitrogen excretion. Some organisms have developed ancillary organs (gills for fish, skin for amphibians, and rectal or orbital glands for sharks, crocodiles, and birds). However, we humans are largely stuck with “body-is-a-box.” Entry is via the mouth and excretion occurs through the kidneys and to a minor extent via the feces.

In 1977, Taylor pointed out that a renin-like material is present in many vertebrate species [13]. He observed no evidence of renin's presence in cartilage fish and concluded that renin first appeared in bony fish, amphibians, and subsequent vertebrates. Nishimura and Bailey published a remarkable early paper on the intrarenal RAAS in various vertebrates [14]. They pointed out that renin activity and granulated epithelial JGA cells are present in bony fish, dipnoans (lung fish), amphibians, birds, and mammals. Nishimura, Bailey, and others observed that in fish, JGA cells are distributed along small arteries and arterioles of the kidney. They also cited evidence that Ang II increases blood pressure in bony fish and that reduced renal perfusion increases plasma renin activity (PRA) in this family. Nishimura and Bailey investigated the toadfish [14]. This animal belongs to the *Batrachoididae* family that includes members without glomeruli (like the seahorse). They performed a remarkable study of blood pressure reduction in the toadfish and reported the resultant effect on PRA. The PRA assay measures the conversion of angiotensinogen to Ang I and is *not* the same as measuring renin directly. However, the responses were not different than we would expect in mammals. Nishimura and Bailey also studied the effects of captopril, an ACE inhibitor, and proved that the effects involved Ang II generation. The investigators next directed their attention to birds (skipping reptiles). They subjected hens to hemorrhage. They found a *pari-passu* increase in PRA with decrease in blood volume and blood pressure in the fowl. They next infused Ang II in the chickens and showed a remarkable effect on blood pressure and sodium (as chloride) excretion, implying RAAS regulation.

Liang et al. [15] were the first to identify renin genes in two non-mammalian vertebrates, zebrafish (*Danio rerio*) and pufferfish (*Takifugu rubripes*). RT-PCR results confirmed generation of the predicted zebrafish mRNA and its expression in association with the opisthonephric kidney of adult zebrafish. They then performed comparative in situ hybridization analyses of wild type and developmental mutants. Their findings indicated that renin expression was first detected bilaterally in cells of the inter-renal primordia at 24 h post-fertilization. The cells subsequently migrated to lie adjacent to the glomerulus of the developing pronephric kidney. The observation that the earliest renin-expressing cells that arose during ontogeny of a teleost vertebrate are of adrenocortical lineage raises an interesting hypothesis regarding the origin of renin-expressing cells in the metanephric kidney of higher vertebrates. Expression of renin in the adrenal gland of murine embryos indicates that this is also first in ontogeny in mammals and further supports that this renin expression is ancestral to renin's expression in the kidney [16].

Salzet et al. [17] have reviewed RAAS elements in invertebrates and vertebrates. Since genome sequences were not available at the time of their report, their study could not rely on genomic data, but instead included inferences from immunoassays and immunohistochemistry. They recognized the presence of ACE-like proteins in the fly and renin-like enzymes in leeches. They extended their discussion to certain vertebrate models. The authors pointed out that snakes developed anatomical and functional adaptations and interesting structural peculiarities that are found in their autonomic, kallikrein-, renin–angiotensin-, and endothelin-related systems.

The physiological evidence indicates that the RAAS was established in bony fishes. Examining the lamprey could help us establish the origin of the system more precisely since these cyclostomes are the sister taxon of all living jawed vertebrates, the gnathostomes. Brown et al. established a radioimmunoassay to measure Ang II in lampreys [18]. They performed acute volume depletion by removing 40 % of the animal's blood volume. This maneuver doubled Ang II concentrations. They then exposed the animals to a decrease in salinity (758 to 605 mosm/kg H_2_O), which rapidly decreased Ang II with a subsequent increase in Ang II. Injecting saline solution intraperitoneally into fresh-water-acclimated lampreys also decreased Ang II concentrations. The results suggest that Ang II may play a role in volume regulation of these primitive vertebrates. The data are consistent with the idea that the Ang II peptide has been around for 500 million years. Missing from the authors' data are mass-spectrometry-determined amino acid sequences of the peptides. These results are fascinating; however, more precise methodologies would be important to prove beyond any doubt that the investigators indeed were dealing with Ang II [19].

## DNA sequence analysis of an ancient system

**Fig. 2:**
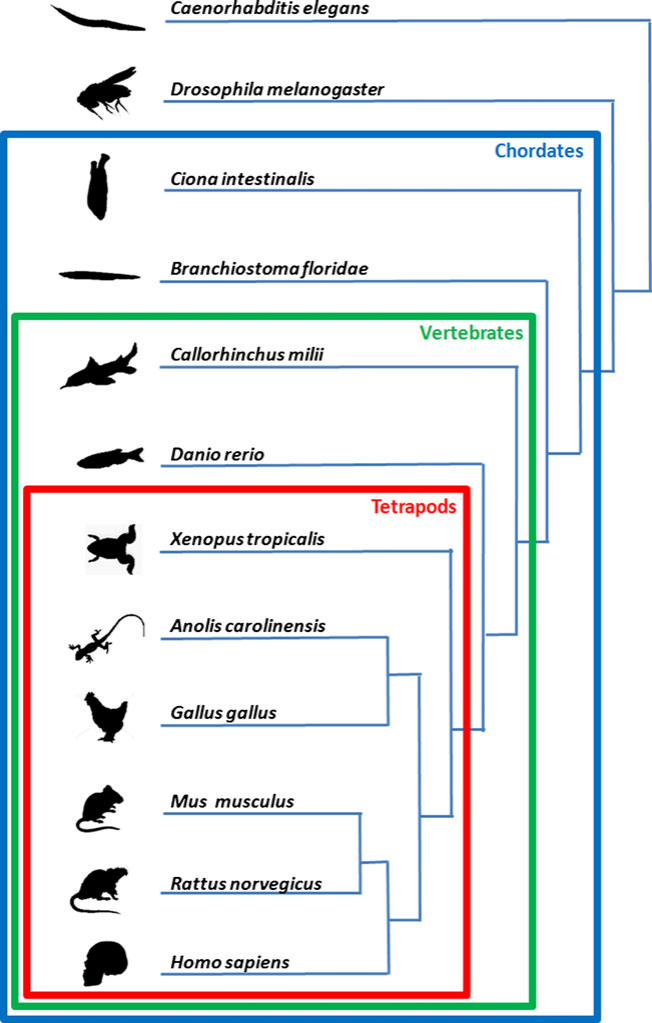
Phylogeny of the species whose sequences are studied in this review. The main groups displayed are the chordates, which comprise all species considered except *Drosophila* and *Caenorhabditis*, and display a notochord, at least at some point during their embryonic development. Vertebrates are species displaying vertebra and comprise all considered chordate species, with the exception of *Ciona* and *Amphioxus*. Finally, the tetrapods comprise all our vertebrate species, with the exception of fishes, either cartilage fishes (elephant shark, *Callorhinchus Milii*), bony fishes (zebrafish, *Danio rerio*), coelacants, or lungfishes

To complement the available physiological evidence on the evolution of the RAAS, we used the information available from the gene and protein sequences in public databases [20]. Complete genomes are especially useful since they allow us to evaluate the presence of a given protein and also inform us of its absence. Particularly, the latter state-of-affairs can be very revealing [21]. We focused our analysis in the search for sequence homologs of genes encoding nine of the human proteins mentioned previously in 12 representative model organisms (Fig. 2 and Table 1). The genomes of these organisms, except the one of the elephant shark, *Callorhinchus milii*, have been completely sequenced. We used the BLAST algorithm [22] to search the NCBI protein database. Orthology was verified with reciprocal searches. Finally, the sequences collected were aligned, and construction of phylogenetic trees was used to verify that the sequences were orthologous to the human gene. We note that although the function of some proteins that we report has been experimentally verified, many are merely predicted translation products from genes resulting from genome sequencing projects awaiting verification. Furthermore, sequencing of additional genomes could clearly make this analysis more complete.

**Table 1:**
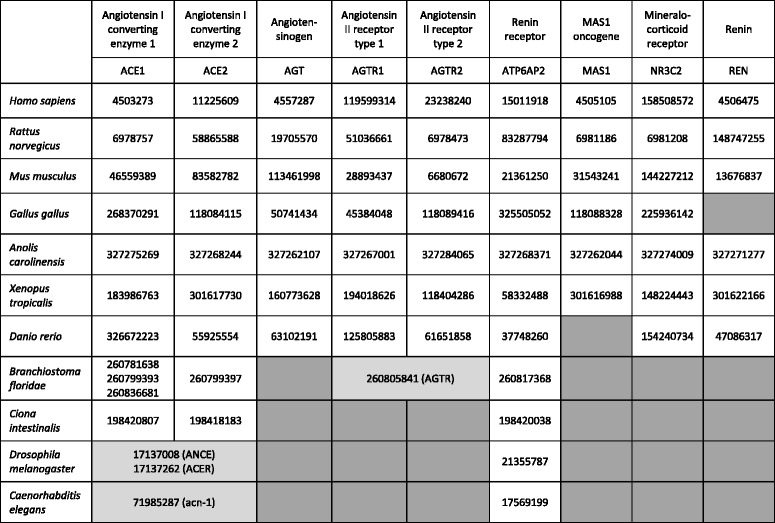
Homologs of nine human proteins of the renin–angiotensin–aldosterone system. Sequences are identified by GenPept identifiers

Empty cells are highlihgted in dark gray to remark that we did not find the corresponding ortholog in the indicated species. Cells shaded in light gray indicate genes that are ancestral to multiple human genes

**Fig. 3:**
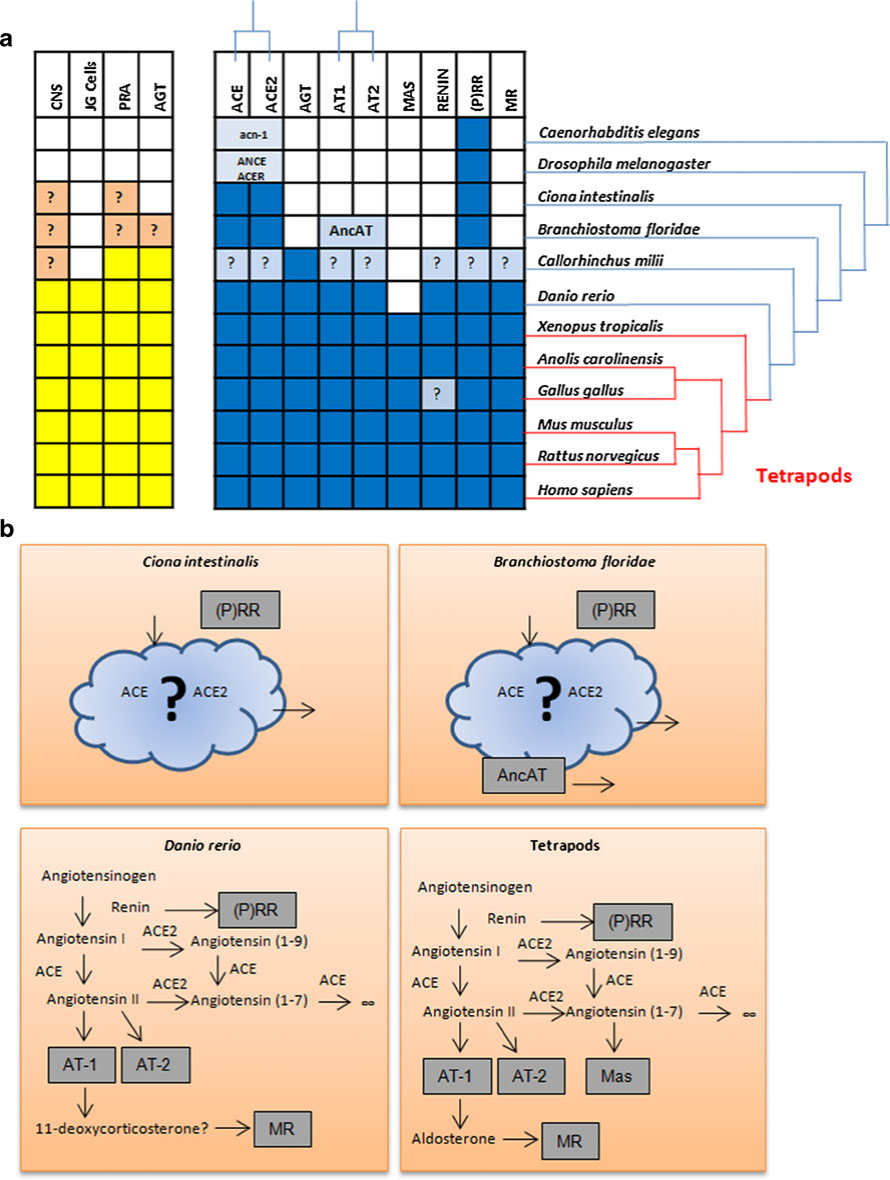
Comparison of the RAAS in multiple species. **a** *Left panel* (*yellow*) includes data from physiological studies: presence known or supposed of RAAS in the central nervous system (CNS), juxtaglomerular cells (JGCells), plasma renin activity (PRA), and angiotensin or angiotensin-like activity known (AGT). Note that PRA does not measure renin, but rather the conversion of AGT to Ang I; renin is not the only enzyme with this capability. *Right panel* (*blue*) shows sequence data found by BLAST inquiry. *Blanks* indicate instances in which the property could not be found. *Question marks* denote instances of uncertain or contradictory data. **b** Model of the stepwise emergence of the components of the RAAS based on their conservation across several taxonomic divisions. *Ciona intestinalis* contains the two ACEs and the prorenin receptor, but the many components missing show evidence that these three proteins have functions ancestral to the RAAS. *B. floridae* has an additional member, AncAT, an ancestral version of the angiotensin receptors. After a large gap, our next closer relatives whose complete genomes we know, the bony fishes, have a human-like system, with two notable differences: a possible use of a precursor of aldosterone and the absence of the Mas receptor. The tetrapods (mammals, reptiles, amphibians, birds) have the complete system, with the exception of renin, which could be missing in aves (see main text)

The main pattern of our findings agrees with physiological evidence (Fig. 3). Most of the components emerge concomitantly to appearance of the juxtaglomerular apparatus. For instance, the zebrafish, *Danio rerio*, our representative of bony fishes, constitutes the taxon with the most primitive JGA [23] and already contains orthologs to eight of the nine proteins considered. The exception is the oncogene Mas, which seems to have evolved later in the tetrapod lineage. We found Mas in the frog *Xenopus tropicalis*, our amphibian representative. Orthologs of the nine genes could be found in all tetrapod species analyzed, indicating the molecular stability of the pathway. We could not find the renin protein in *Gallus gallus*, the chicken, representing birds in our study. However, clear homology to full human renin was found in genomic shotgun sequences corresponding to chromosome 26 of *Gallus gallus*. These sequences have not been yet assembled to the current version of the *Gallus gallus* genome.

Angiotensinogen, because of its central role in the system, could be the best molecule to establish the time of RAAS emergence. We found an angiotensinogen ortholog in the shark *Callorhinchus milii*, confirming that the RAAS was established with the appearance of gnathostomata, the jawed fishes. Unfortunately, the shark genome is not yet completely sequenced, and we could not find any other sequences of the genes we tested.

We could not find an angiotensinogen ortholog in lampreys. We note that some lamprey sequences are currently annotated in GenBank as putative angiotensinogens (e.g., FM954978 from *Lampetra fluviatilis* [24]), but reciprocal sequence similarity searches to the proteins from bony fish *Danio rerio* or human suggest that they are orthologs of SERPIND1.

**Fig. 4:**
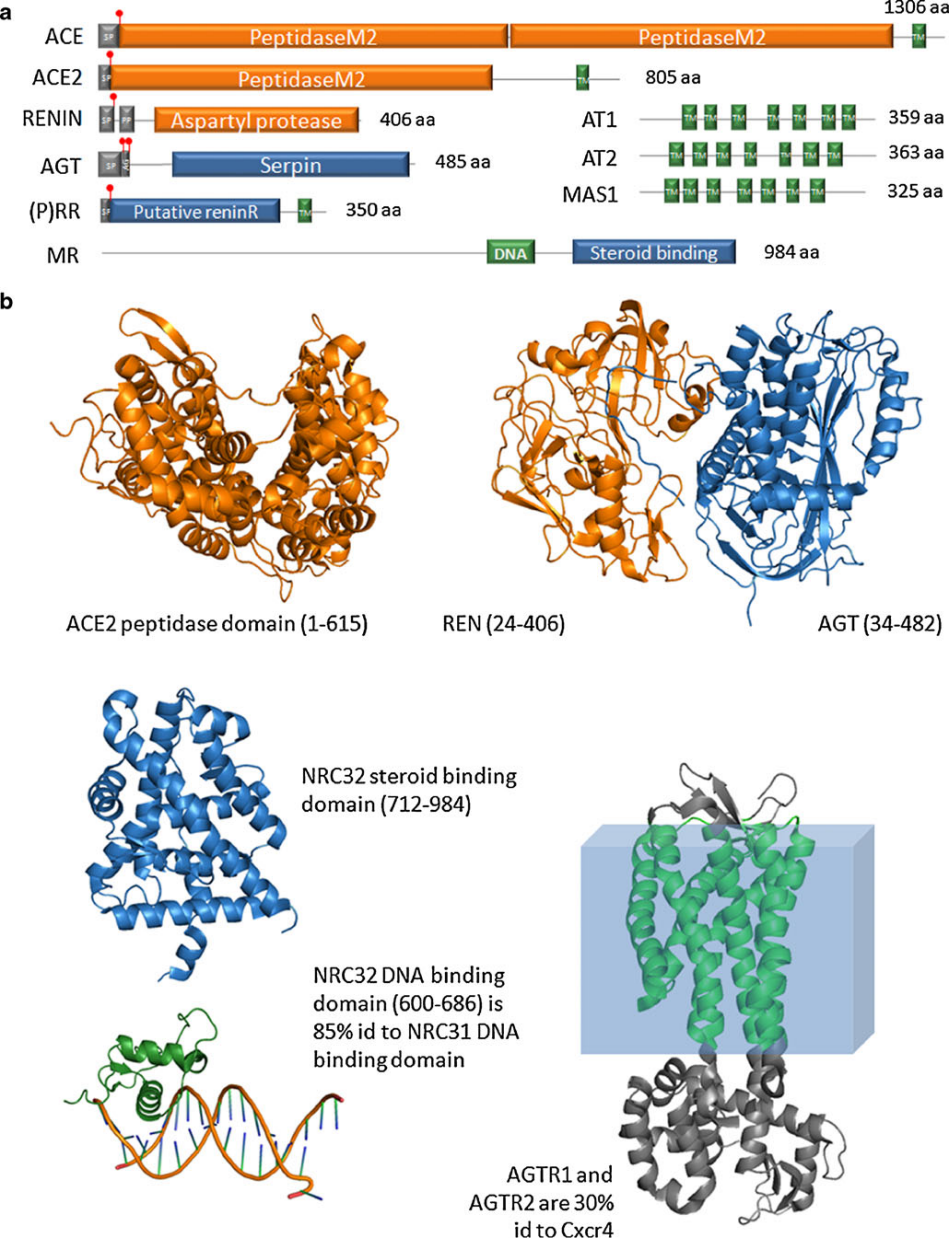
Structural features of nine human proteins relevant to the RAAS. **a** Domain organization of ACE, ACE2, renin, AGT, (P)RR, and MR. *T* transmembrane alpha-helix (TM), *S* signal peptide (SP), *P* pro-peptide (PP). *Red box* on angiotensinogen diagram: Ang I sequence (AG). *Red symbols* indicate protein cleavage sites. **b** Solved 3D structures of these proteins or homologs (when indicated). ACE2: peptidase domain (fragment 1-615, PDB:1R42); REN and AGT: complex of renin (*blue*) and AGT (*orange*). Note the N-terminal of AGT protruding into the renin molecule for processing (PDB:2X0B); NRC32: steroid binding domain (*blue*; PDB:2AA2) and DNA binding domain (*green*) with DNA (stick model) from 85 % identical rat glucocorticoid receptor NRC31 (PDB:3G9P); AGTR1/AGTR2 are 30 % identical to the CXCR4 chemokine receptor whose structure is shown (TM helices in *green*; PDB:3OE0). All protein structures are represented using the PyMOL Molecular Graphics System software (DeLano Scientific, Palo Alto, California)

Angiotensinogen displays a serpin domain (Fig. 4a). Serpins are known as protease inhibitors [25]. Ancestral binding of angiotensinogen to proteases may have been a prerequisite to cleavage of angiotensinogen by proteases such as renin [26].

The fact that some of the genes analyzed existed prior to the appearance of angiotensinogen can be used to trace the ancestry of the system. However, the finding also indicates the incompleteness of our functional understanding regarding these genes. The best example of the latter conclusion can be illustrated with the mysterious (P)RR, also known as ATP6AP2. We observed (P)RR orthologs in all organisms with complete genomes analyzed, including *Drosophila melanogaster*, our insect representative, and the worm *Caenorhabditis elegans*. The sequence length was fairly constant, suggesting a well-conserved domain organization. ATP6AP2 presumably gained function with respect to the binding of prorenin and renin, later in evolution [7]. Most likely, the more ancestral function of this protein is related to its association with vacuolar H(+)-ATPase, a complex that regulates the pH of several intracellular compartments. Whether or not these two seemingly unrelated functions are interrelated remains to be determined [27].

Evidence of functions ancestral to the emergence of the RAAS can also be proposed for ACE and ACE2. We found orthologs of both proteins in *Ciona intestinalis*, the sea squirt, which is our tunicate representative and in *Branchiostoma floridae*, the amphioxus; both representing primitive chordates. These two model organisms lack most of the molecular and physiological components of the system, including angiotensinogen. ACE and ACE2 are absent in *Drosophila*, which has homologous sequences sharing probable ancestry but that belong to a different family, namely, the angiotensin converting enzyme gene (ANCE) and angiotensin converting enzyme related (ACER). Both of these enzymes are active endopeptidases during fly development but seem to lack a clear function in adulthood [28].

ANCE is able to hydrolyze Ang I [29], whereas ACER cannot perform this function [28]. This finding suggests that the enzymatic activity for the processing of angiotensinogen products existed prior to the appearance of angiotensinogen itself. Angiotensinogen could possibly have emerged as an adaptation to existing ACE metalloproteinases.

It is interesting to note that ACE activity seems to be related to fertility and development in organisms ranging from insects to mouse. ANCE has been suggested to play a role in the peptide-processing enzyme in seminal fluid of *Drosophila* [30]. In the mosquito *Anopheles stephensi*, ANCE may regulate embryogenesis when activated by blood meal [31], while in freshfly *Neobellieria bullata*, several substrates or inhibitors of ACE activity are present during development of ovaries, suggesting a role of ACE activity in the reproductive system [32]. Besides, an isoform of ACE is only present in germ cells of wild-type male mice [33], while male mice deficient for germinal ACE show infertility [34].

**Fig. 5:**
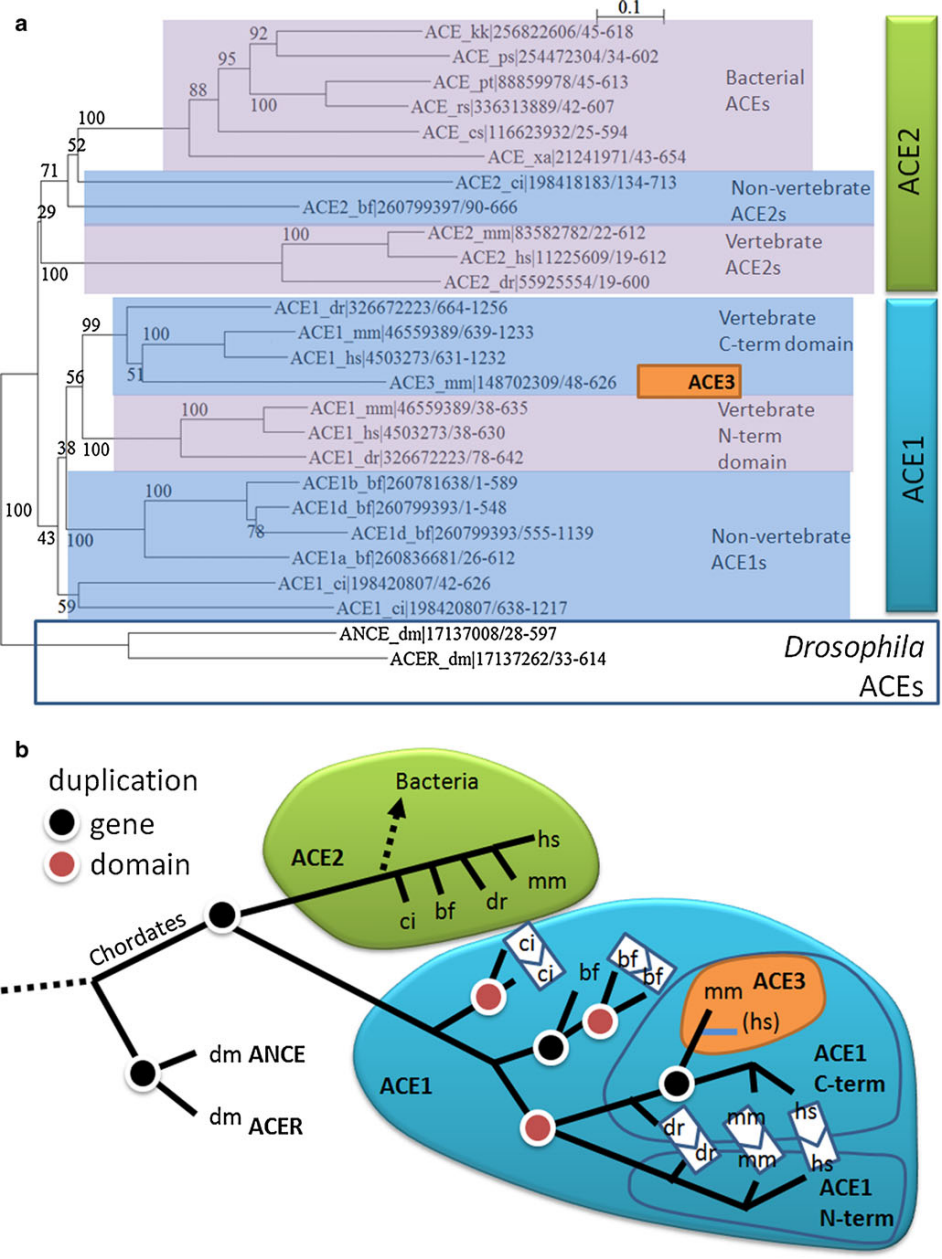
Evolution of the ACE family. **a** Phylogenetic tree of the peptidase domains of selected eukaryotic and bacterial ACE homologs. The numbers at the branches indicate number of bootstrapping tests that resulted in the marked grouping: Values close to the total used (100) indicate reliable branches. The labels indicate the subfamily, a two letter abbreviation of the species name, GenPept identifier, and amino acid range. Species abbreviations of eukaryotic species are dm (*Drosophila melanogaster*), ci (*Ciona intestinalis*), bf (*B. floridae*), dr (*Danio rerio*), mm (*M. musculus*), and hs (*H. sapiens*). ACE_xa corresponds to the bacterial *Xanthomonas axonopodis* sequence; for the other bacterial species, please refer to the database records. Drosophila sequences contain a single domain (ANCE_dr, ; ACER_dm) and constitute an outgroup indicating that they are ancestral to chordate ACE1/ACE2. Multiple bacterial sequences (including the *X. axonopodis* sequence) contain a single protease domain that groups with single domain ACE2s and is not ancestral to both ACE1 and ACE2. This suggests that the bacterial sequences are a result of horizontal transfer from an ancestral chordate species. **b** Interpretation of the phylogenetic tree. The ACE family originated before the divergence of chordates from arthropods. Gene duplications (*black dots*) have expanded this family, for example, leading to the existence of ACE1 and ACE2 in chordates. Multiple events of domain duplication (*red dots*) have happened in the ACE1 subfamily, an important one leading to the vertebrate ACE1, which contains an N-terminal and a C-terminal catalytic domain. ACE3 is a single domain ACE, which stems from duplication of the mammalian C-terminal domain of the ACE1. This sequence seems to have evolved into a pseudogene in humans (*blue line*). Orthologs of vertebrate ACE2 are present in many bacterial species. Their close homology to non-vertebrate ACE2s suggests that they are the result of a single event of horizontal transfer from an ancestral non-vertebrate species. The grouping in the phylogenetic tree of the bacterial sequences analyzed here suggests that this initial event was followed by further events of horizontal transfer between bacterial species, indicating that bacterial ACEs have acquired a function that confers an evolutionary advantage to the species bearing it. Multiple sequence alignment was produced using the MUSCLE method [55] as implemented at the EBI web server. The alignment was examined, and phylogenetic trees were generated using ClustalX Version 2.1 [56] excluding positions with gaps and correcting for multiple substitutions

The biological plasticity in *repurposing* the catalytic domain of the ACE enzymes is further exemplified by the fact that many bacteria contain an ACE protein that cleaves Ang I to Ang II. This state-of-affairs was proven for the *Xanthomonas axonopodis pv. citri* protein [35]. Interestingly, we observed that the closest homologs of this bacterial sequence in eukaryotic species are non-vertebrate chordate orthologs of human ACE2, for example, *Branchiostoma floridae*, GenPept identifier 260799397 (E value = 1e-168). More significantly, our phylogenetic analysis clusters bacterial sequences with the ACE2 sequences of *Ciona intestinalis* and *Branchiostoma floridae* (Fig. 5). Our observation suggests that this bacterial family is the result of a horizontal transfer event involving an ancestral ACE2 that happened in an ancestor of *Ciona intestinalis* after the divergence of tunicates from other chordates. The structure of the bacterial phylogenetic tree suggests further events of horizontal transfer involving this gene between bacteria. Besides their enzymatic properties, the ACEs have other non-catalytic functions [36]. Bacteria might exploit these other functions as well.

Domain analysis of this family is interesting because the ACE2 orthologs (including the bacterial ones) and *Drosophila melanogaster* ANCE and ACER possess only a single 600 amino-acid peptidase domain (peptidase M2 domain). In contrast, ACE orthologs have two such domains ([37], Figs. 4a and 5). The strong similarity between the N- and C-terminal domains in one *Ciona intestinalis* and in one *Branchiostoma floridae* sequence suggest that the proteins of the ACE subfamily have undergone multiple independent events of domain duplication like the one leading to the vertebrate ACEs. In mice, the two domains have different importance in the development of hypertension, as has been shown by selective deletion of one of the two domains [38].

Finally, in mammals, we observed a further member of this family containing a single catalytic domain, termed ACE3. The function of ACE3 is unknown. The best characterized member is the mouse ACE3, which is expressed and produces a protein in sperm; however, gene deletion does not render mice infertile [39]. Orthologs of ACE3 exist in cow, dog, and rat, while the human counterpart is a pseudogene with multiple base deletions, insertions, and stop codons. Moreover, murine ACE3 lacks the residues necessary for ACE catalytic activity [40]. ACE3 expression could possibly regulate the expression of the parental ACE gene, a mechanism of post-transcriptional gene regulation that is known to exist for other pseudogenes [41]. The position of the ACE3 gene in human and mouse genomes downstream from ACE is consistent with this view. Our sequence analysis indicates that ACE3 is the result of an N-terminal domain duplication of ACE (Fig. 5). We did not find orthologs of this gene outside of mammals.

It is important to note that beside ACEs, other enzymes that can cleave Ang I to Ang II [5] have been found, such as chymase, a protein expressed in the mast cells of heart and blood vessels [5], and cathepsin G, a secreted serine peptidase of neutrophils and mast cells [42]. These proteins appear to have evolved well after the establishment of the RAAS.

The AT_1_ and AT_2_ receptors, also termed AGTR1 and AGTR2, are also evolutionary products of gene duplication. We identified an ancestral sequence of the two proteins in *Branchiostoma* (AncAT, Fig. 3a; supplementary Figure 1). We did not find any homologue in the most distant *Ciona.* This finding suggests that this protein family emerged after divergence of tunicates from primitive chordates. Again, the functional relationship of this gene with Ang II must have occurred later in evolution since angiotensinogen evolved later (Fig. 3b). The Ang II receptor family may exhibit other ancestral functions.

The MR probably predated angiotensinogen and renin. The gene for the ancient MR protein might have been duplicated in an ancestral organism before the emergence of bony fishes [43, 44]. The primordial MR then evolved (duplicated) to become a glucocorticoid receptor (GR). This interpretation suggests that volume regulation is older than stress-related responses. We traced MR easily to bony fishes. Our cartilage fish sequence left us with a question mark (Fig. 3a), and we will have to await additional sequence information to be able to establish more precisely the time of emergence of this receptor.

Finally, some inbred strains of laboratory mice (not the widely used C57Bl/6) harbor a second renin gene (Ren-2) [45]. Ren-2 is mainly expressed in the salivary gland of males and is dramatically stimulated by aggression (100,000-fold higher levels in saliva than plasma) [46]. Possibly, the aspartyl protease serves to injure a bitten opponent or conceivably the protease serves a protective function for wound healing after a fight by wound licking. Interestingly, increasing angiotensinogen in the mouse by infusion increases blood pressure substantially. The normally fairly low levels of angiotensinogen in the blood of mice could be a response to the effectiveness of their salivary renin. The fact that Ren-2 is strain-specific suggests that the duplication of this gene is a relatively recent event. A relative paucity of renin substrate in the mouse could have favored this duplication [47]. Ren-2 actually provided the first successful transgenic-rat model of hypertension, underscoring its amazing effectiveness in cleaving rat (cross species) angiotensinogen [48].

## Discussion

The important finding of our study is that most of the RAAS component members began to appear with primitive chordates and tunicates. All of the important components were present with the development of bony fishes, with the exception of the Mas receptor, which appears in amphibians. We found solid evidence that angiotensinogen made its appearance in cartilage fishes, but they are still a mystery as this is the only sequence of the RAAS available so far in sharks. This situation is unfortunate as this taxon is of crucial importance in the understanding of the evolution of volume regulation.

We included the (P)RR in our analysis, although the protein has no known function in volume regulation. The receptor has a component that serves as an important adapter protein for a vacuolar ATPase, and for this reason, the sequence is probably much older than the RAAS-related function [7]. The function of this receptor is still not clear, so the time point when its interaction with prorenin and renin developed remains speculative.

**Fig. 6:**
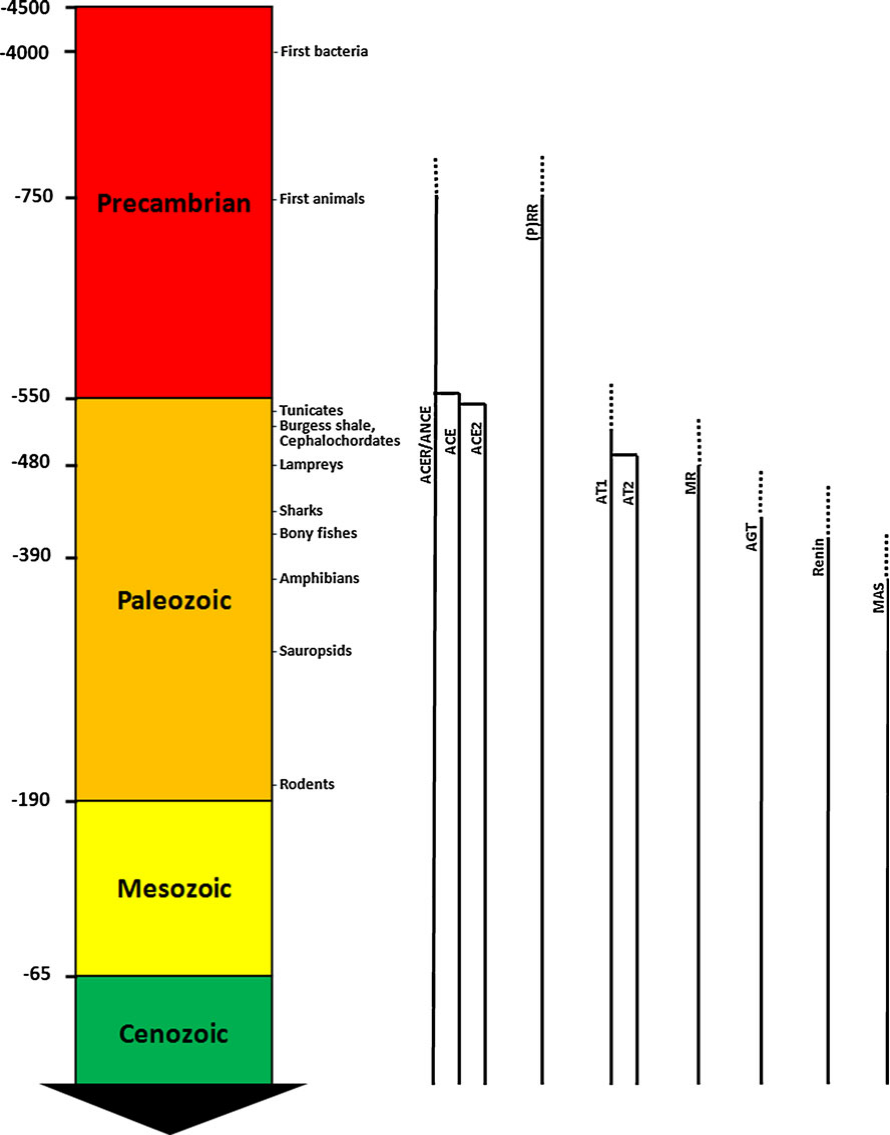
Time-line of the emergence of the RAAS. *Left* geological eras and a time-line (scale in millions of years). While most genes appeared in the early Paleozoic, others might have emerged earlier in the Precambrian era and were adapted for their use as part of the RAAS. ACE is one such example and might have evolved from an initial developmental function to physiological actions on volume regulation in vertebrates

We suggest a time-line for the evolution of the RAAS components from Precambrian times to the present (Fig. 6). Our scheme suggests that the emergence of the RAAS resulted from the buildup of a peptidase core and subsequent receptors working on an unknown substrate (cloud in Fig. 3b). This core would have adapted to the processing of angiotensinogen, was placed under the control of renin, and finally completed its aim of volume regulation with some adjustments in the downstream receptor elements. These elements included the Mas oncogene and the MR. The MR probably uses 11-deoxycorticosterone, the precursor of ALD, in fishes, instead of ALD [49]. This entire process would have occurred along 150 million years of evolution in Paleozoic times followed by 400 million years of relative stability. Our findings confirm and extend previous studies of the evolution of the RAAS, which point to its emergence 400 million years ago, about the time cartilage and bony fish diverged [13].

We are aware of shortcomings in our study. The vertebrate classes are exceptionally diverse. Among them are sharks that live in fresh water, bony fishes that spend time in fresh water and then in sea water and vice-versa, sea-going frogs and crocodiles, birds that fly over 5,000 km water without any fresh water, and mammals that survive without or within, fresh or sea, water. How do they do it? We have merely scratched the surface. The transition from genomic data to function, pathophysiology, and relevance for man in health and disease is long and still incomplete. We believe that it may be rewarding in the future. The RAAS is a relatively well-studied system for these aspects. We would be curious how Homer Smith would view these genomic data. He would have probably demanded that the lungfish be sequenced. We agree, and the more sequence data we have, the more predictions we could make and the more likely we will able to eventually identify the molecular events that led to the interaction of relatively new ligands (renin, angiotensinogen) to much older proteins that had earlier different functions (ACE, AT_1_, AT_2_). However, the data available provide ample evidence that new fields of research, such as genomic, molecular evolution, and evolutionary medicine, are emerging. This state-of-affairs has been made possible by methodological advances in sequencing technology and internationally available, annotated databanks. With these new tools and knowledge, new hypotheses can be formulated and tested computationally and experimentally. The questions why specific genes and metabolic pathways have been of advantage for survival and reproduction during evolution can be answered by detailed molecular analysis. A highly active RAAS offered an evolutionary advantage because salt and volume homeostasis were important for survival. Dramatic changes in environment when moving from salt water to fresh water (fish), then to combined habitat water and land (amphibians) and to land (reptiles, primates), had to be met by an efficient regulatory system to maintain homeostasis. Such a condition was true until *Homo sapiens* appeared 150,000 years ago [50] and beyond. With the invention of salt for conservation in stored food in advanced civilizations and excessive salt content in industrialized nutrition, the RAAS is in constant *overdrive*, causing salt and volume overload in the body with ensuing hypertension, stroke, and cardiovascular diseases. There is overwhelming evidence that salt is an important cause of hypertension [51, 52]. The gap between the early function of the RAAS and the completely different environmental challenges and nutrition today may be one of the evolutionary reasons why hypertension, the number-one risk factor for mortality worldwide, occurs in about half of the adult population nowadays [53].

In conclusion, by studying evolution, we can gain insight into how our body works and eventually obtain clues on how to deal with dysfunctions. We suggest that the sustaining efforts in better understanding the RAAS will have soon importance in the study of hypertension and other cardiovascular diseases. These would be important studies to advance the concept of an evolutionary medicine [54].

## Electronic supplementary material

Below is the link to the electronic supplementary material.

Figure S1 Phylogenetic tree of the AT_1_ and AT_2_ receptors. The two main branches correspond to the orthologs of AT_1_ (AGTR1) and AT_2_ (AGTR2) in vertebrata. The outlier represents a sequence ancestral to both families in *B. floridae* indicating that they are the result of an event of gene duplication after the divergence of cephalochordata from vertebrates. The labels include the GenPept identifiers and other information regarding the sequences used. The phylogenetic tree was compiled as explained in the caption of Figure 5. (JPEG 131 kb)

High Resolution Image (TIFF 3163 kb)
