# Supplementary figures and images for: Emergence and evolution of the renin–angiotensin–aldosterone system
Source: J Mol Med (Berl). 2012 Apr 14;90(5):495–508. doi: 10.1007/s00109-012-0894-z (PMC3354321; doi:10.1007/s00109-012-0894-z)

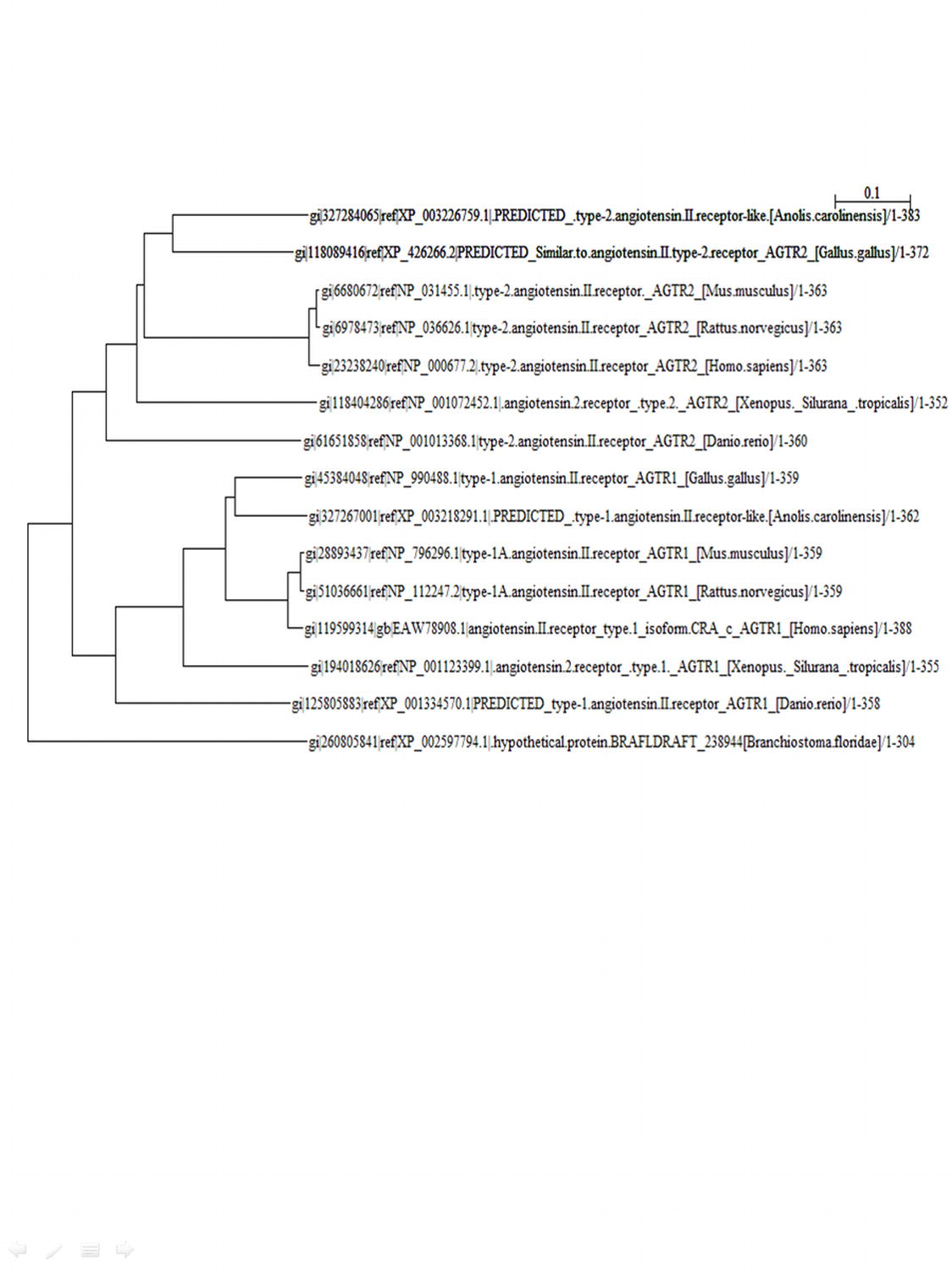

Supplement: Supplementary file 1 — Phylogenetic tree of the AT1 and AT2 receptors. The two main branches correspond to the orthologs of AT1 (AGTR1) and AT2 (AGTR2) in vertebrata. The outlier represents a sequence ancestral to both families in B. floridae indicating that they are the result of an event of gene duplication after the divergence of cephalochordata from vertebrates. The labels include the GenPept identifiers and other information regarding the sequences used. The phylogenetic tree was compiled as explained in the caption of Figure 5. (JPEG 131 kb) [file 109_2012_894_Fig7_ESM.jpg]

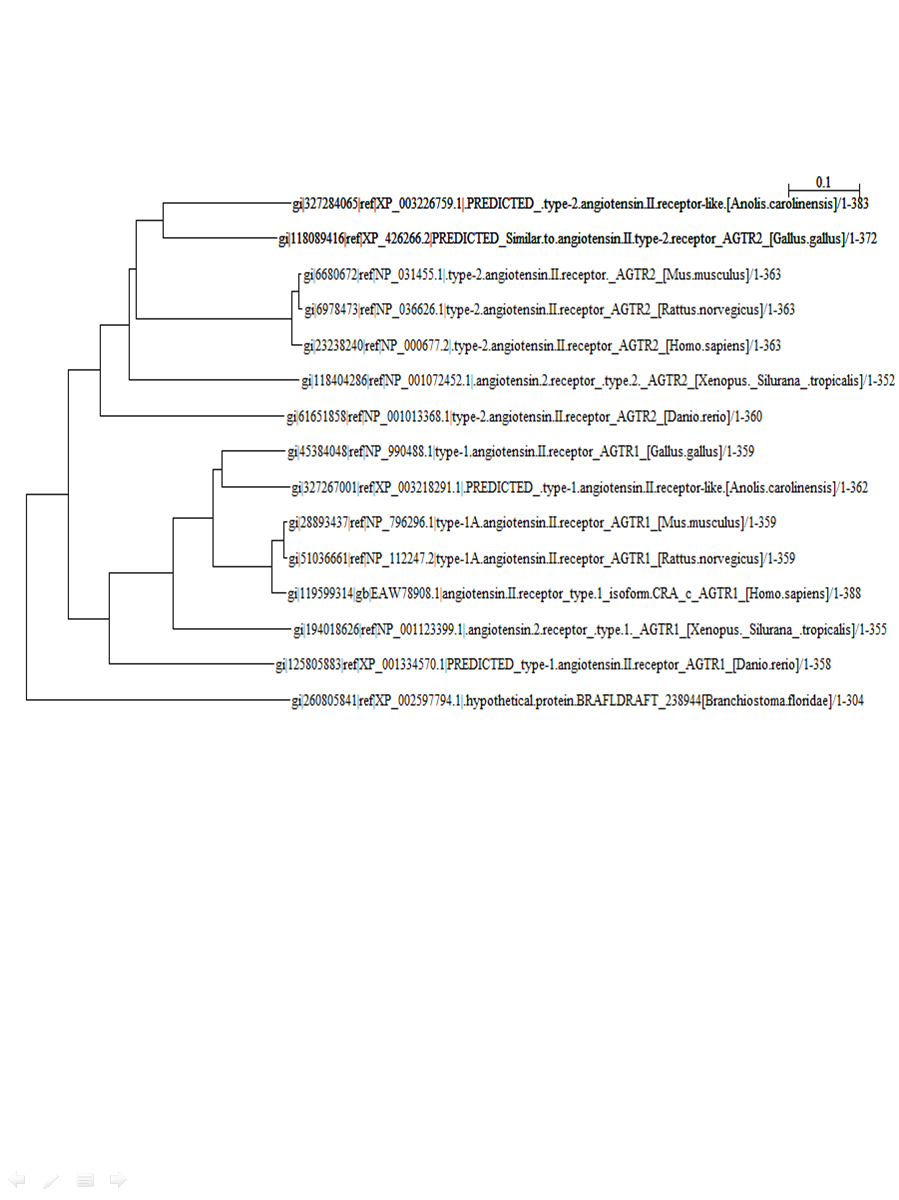

Supplement: Supplementary file 2 — High Resolution Image (TIFF 3163 kb) [file 109_2012_894_MOESM1_ESM.tif]
